# Combination of Phagostimulant and Visual Lure as an Effective Tool in Designing House Fly Toxic Baits: A Laboratory Evaluation

**DOI:** 10.1371/journal.pone.0077225

**Published:** 2013-09-24

**Authors:** Hafiz Azhar Ali Khan, Sarfraz Ali Shad, Waseem Akram

**Affiliations:** 1 Institute of Agricultural Sciences, University of the Punjab, Lahore, Pakistan; 2 Department of Entomology, Bahauddin Zakariya University, Multan, Pakistan; 3 Department of Entomology, University of Agriculture, Faisalabad, Pakistan; Fundação Oswaldo Cruz, Brazil

## Abstract

House flies (Diptera: Muscidae), potential vectors of a variety of pathogens, characteristically search and feed on sugar sources just after emergence for their survival. Phagostimulants like sugars, and visual characteristics of feeding materials play an important role in foraging success in house flies. Therefore, development of toxic baits by using the combination of phagostimulant and visual lure may prove effective in localized control of house flies. In the present study, visual attraction of house flies to different fabric colors was studied in choice and no choice experiments. Dark blue was the most preferred color in both experiments. In toxicity experiments, insecticide solutions were prepared in 20% sugar solution. Dark blue fabric strips were prepared by moistening with 20% sugar water solution containing median lethal concentrations of one of the four insecticides viz., fipronil, Imidacloprid, indoxacarb and Spinosad. The fabric strips treated with fipronil and Imidacloprid took minimum time (7.66 and 7.81 h, respectively) to cause 50% mortality, while those treated with Spinosad and indoxacarb took relatively more time (13.62 and 17.91 h, respectively) to cause 50% mortality. In conclusion, the combination of phagostimulant and visual lure could be used in designing toxic baits for house flies.

## Introduction

House flies, *Musca* *domestica* , are among the major ecto-parasites of livestock that have the potential to transmit a number of diseases in urban and rural settlements [1]. Chemical control has been considered as a key component in the management of this notorious pest; however, the development of insecticide resistance and environmental hazards create the demand to explore alternate methods and/or modifications in chemical control measures [2-5]. Localized toxic target technique, like insecticide application through baits, is advantageous as it not only reduces the risks of insecticide resistance development in flies, but also reduces the amount of toxicant released into the environment [6]. Since insecticides used in baits are localized in nature, attraction factors should be explored to make the baits successful in the house fly management [7]. The success of toxic baits may depend on many factors like the use of phagostimulants, visual and/or olfactory lures to attract and ultimately kill fly populations. Among phagostimulants, sugar is a potential factor in attracting house flies to the baits because it provides critical nutrients for their survival [8]. Adult flies emerge with little stored energy [9] and they have to find sugar sources and water for their survival [8]. This predisposition of house flies to seek sugar sources for their survival presents an opportunity to use sugar in toxic baits. For this reason, sugar along with toxicant, water and/or other attractant, in many other studies, has been an essential component in making toxic baits successful [6,10-13].

The role of visual lures in attracting house flies to toxicants has not been studied to much extent in the past [14]. Visual lures have been considered as important stimuli in determining changes in house fly behavior such as attraction and/or repulsion, since much of the head is occupied by two compound eyes, and a cluster of three ocelli [15]. These eyes receive reflected light and send the message to the nervous system where optic lobe interprets the message and may elicit the response in the form of attraction or repulsion [16]. Different colors have been employed in making fly traps attractive, but the data on flies’ preferences for different colors are confusing [17], and this might be due to the different spectral ranges of the materials used. Yellow, for example, was found to be most attractive to house flies when used in jug traps [18]. In another study, black was found to be the most attractive to house flies [13], however, Geden [14] reported that blue fabric targets were more attractive to house flies than black targets. Although colors have been used in making traps attractive, studies on the use of colors, to the best of the authors’ knowledge, are rare in making toxic baits attractive to house flies particularly in Pakistan. House fly baits are usually in the form of granules and sprays, however, both formulations have limitations. Granules, for example, needs frequent applications due to the possibility of becoming covered with manures or other debris, particularly in agricultural settings [10]. On the other hand, sprayable baits are also difficult to apply in urban and rural settings due to the possibility of clogging the dispenser with dust particles [12]. In the past, insecticide treated cords had been an effective tool to control house flies [19,20], but their use was interrupted, mainly because most of the organochlorine and organophosphate insecticides used to impregnate them were banned by the Environmental Protection Agency [8]. Recently, however, the interest in cords treated with less hazardous insecticides has re-emerged [21].

Recent reports on insecticide resistance development in house flies to different insecticides from organochlorine, organophosphate, carbamate, pyrethroid and new chemical classes in Punjab, Pakistan [2,3], stress the need to explore alternate control methods, like toxic baits, by which resistance and environmental contamination are minimized. Therefore, keeping in view the importance of toxic baits, following studies were carried out to: 1) evaluate the preference of house flies towards different colored fabric strips in “choice” and “no choice” experiments; 2) evaluate relative efficacies of different insecticides along with phagostimulant coated on colored fabric strips.

## Materials and Methods

### Biological material

Adult house flies used for experimentation were collected from a dairy farm in Multan (30°12′0″ N, 71°25′0″ E) and brought to the laboratory where they were reared as described previously [1]. The field population was reared for five generations before the start of the experiments. In addition, the population had a low level of resistance compared to a laboratory susceptible strain to the insecticides (below) used in the toxicity experiment. No specific permit was required to collect house fly samples from the dairy farm as it was privately owned and collection was made merely by arrangement with the owner. Since the house fly is not an endangered species, no permission was required from any concerned authority in Punjab, Pakistan. All the experiments including rearing of house flies were conducted with ambient temperature at 27 ± 2°C.

### Visual attraction experiment

Fabric strips of eight different colors: black (λ = 620nm; Hex. value = #000000), dark blue (λ = 469 nm; Hex. value = #0000bf), sky blue (λ = 486 nm; Hex. value = #70b1ff), grey (λ = 543 nm; Hex. value = #d1d2d1), pink (λ = 397 nm; Hex. value = #ffb6c1), red (λ = 618 nm; Hex. value = #e03c31), white (λ = 507 nm; Hex. value = #f8fdfd) and yellow (λ = 584 nm; Hex. value = #fff44f), were used in the attraction experiment. The attraction experiment was carried out in “free choice” and “no choice” fashions by following the methodologies of Ahmed et al. [22] and Diclaro et al. [15], with some modifications. Briefly, for uniform impregnation, the fabric strips (10 cm × 2 cm) were soaked in 20% sugar solution (500 ml) for 2 minutes and dried at room temperature until dripping of the solution stopped. In the free choice arena, the strips of all colors were attached with a wooden stick and hung in the middle of the screen mesh cages (40 × 30 × 30 cm), with a 3 cm strip to strip distance. The order of the strips on the wooden stick in each replicate of the free choice experiment was determined by using a random number table. In the no choice arena, the fabric strips of each color were hung separately on a wooden stick in the middle of the mesh cages (1 strip/cage). Before each experiment, 3- to 5-day-old flies were aspirated from the breeding cage and placed in a freezer (-2 °C) until inactive. The flies were then sexed and allowed to recover for an hour before the start of the experiment. Before starting the experiments, 100 female flies were starved for 3-4 hours and then introduced into the experimental cages. The number of house flies resting on a specific colored fabric was observed every 15 min for 2 h. The observations were made very carefully, so that there would be no disturbance to flies during observations. For this, the observer took his position outside the cage 2 minutes before the start of each observation. Although the lighting around the cages was the same as in the experimental room [one light bulb (Philips, Model # 929676000902) in each corner of the room and one in the middle of the room], even then the cages were rotated at 45° after each observation to rule out the possibility of position and light effects. Both the experiments were replicated eight times under continuous light on separate days using different flies.

### Toxicity experiment

Four insecticide solutions viz., fipronil (Regent^®^ 36EC, Bayer Crop Sciences), imidacloprid (Confidor^®^ 20SL, Bayer Crop Sciences), indoxacarb (Steward^®^ 15SC, DuPont) and spinosad were prepared at their median lethal concentrations [3] in 20% sugar solution. Recently it has been reported that some of the dairy farmers in Punjab, Pakistan used these insecticides for the management of flies [3,23]. For the toxicity experiment, the most attractive dark blue fabric strips, from the visual attraction experiments, were treated with a specific insecticide solution and were hung in separate mesh cages in the same way as was used in the no choice arena (see above). Whereas the dark blue fabrics treated with 20% sugar solution without toxicant were used in controls. Twenty 3-5-day-old female house flies were introduced in each mesh cage containing an insecticide treated fabric strip, and control cages as well. The experiment was replicated three times. The toxicity of insecticide treated fabric strips was evaluated by calculating the median lethal time (LT_50_) values. For this, the data were recorded at 1, 6, 12, 24 and 48 h intervals after the introduction of flies to the experimental cages. The flies were considered dead if they were ataxic/unable to move or fly.

### Data analyses

For the visual attraction experiment, the mean number of house flies per specific colored strip was analyzed by a one-way analysis of variance (ANOVA) using the software Statistix 8.1v [24] and means were compared with Tukey’s Honestly Significant Difference (HSD) test. To calculate median lethal time (LT_50_), the toxicity bioassays data were analyzed by Probit analysis using the software SPSS version 10.0. LT_50_ values were considered significantly different based on non-overlapping of 95% confidence intervals.

## Results and Discussion

**Figure 1:**
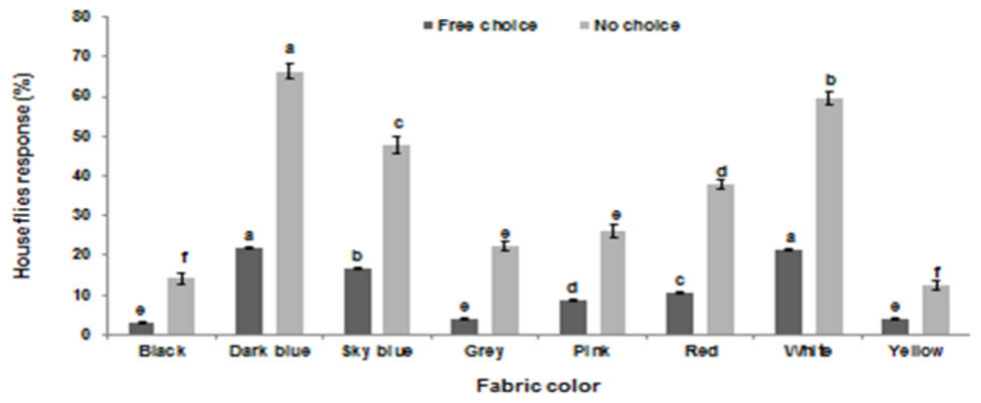
Preference of house flies to the different color fabric strips in free choice and no choice experiments. Bars are mean percent preference (±SE) of house flies. Bars of specific experiment sharing the same letters are statistically at par (Honestly significant difference [HSD] test, Statistix 8.1).

**Figure 2:**
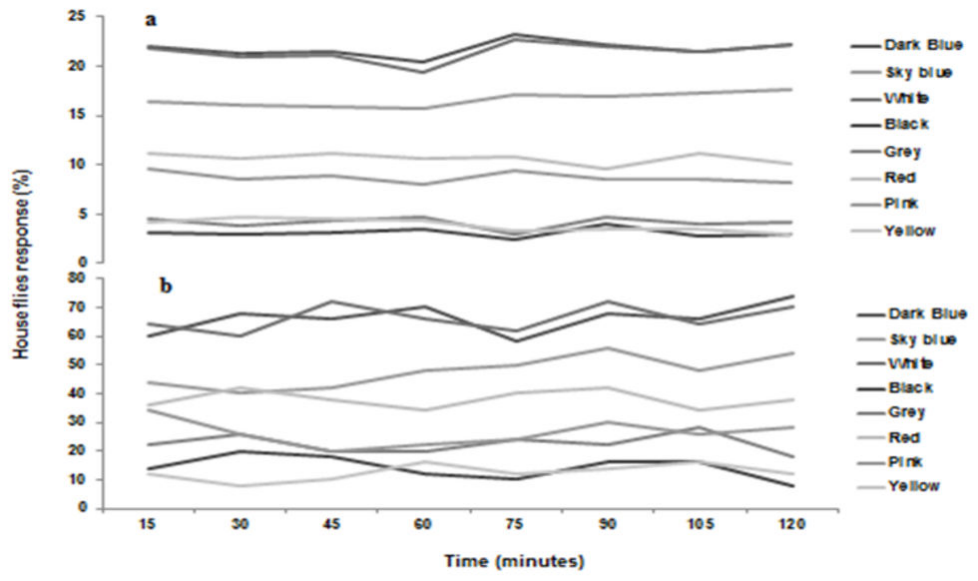
Response of house flies to different color fabric strips over a period of 2 hours with 15 min intervals.

The fabric colors had significant impact on the preference of house flies in both types of experiments (F=1020; df=7,56; P<0.01 for choice experiment, and F=207; df=7,56; P<0.01 for no choice experiment). In the free choice experiment, the highest numbers of resting flies were found on dark blue and white, both were statistically at par, followed by sky blue and red, while yellow and black were least preferred colors. Similarly, in no choice experiment, dark blue was the most preferred one followed by white, while black and grey were the least preferred colors by house flies (Figures 1, 2).

The colored fabrics played an attractive role towards house flies. In both attractiveness experiments, blue and white attracted more flies compared to the rest of the colored fabrics. Male and female flies have equal behavioral and physiological responses towards different color targets [15]. Previously, different colors have been used as a visual attractant in designing fly traps [14,25], but studies on the use of color as attractant material in toxic baits are rare. Our study confirms the findings of Diclaro et al. [15] and Geden [14] who reported that house flies have a significant attraction towards blue colored fabric and plastic materials respectively, and hence concluded that these colors could be used effectively in monitoring activities. They also concluded that the color of visual targets is more critical in attraction than the material of the visual targets, which has no influence in attracting flies. Blue has also been found to be an attractive visual target for other dipteran species [26]. For example, biting flies or tsetse flies [27,28] and stable flies [29] have also shown a preference towards blue colors used in fly traps. However, our study contradicts with the findings of Ahmed et al. [22] who reported that black attracts more flies compared to other colors used in the fabrics. Given the observed attraction of house flies to this color, dark blue could be used effectively in designing baits particularly in warm climates, as it may represent a shaded and cooler resting place for flies [27].

The dark blue fabrics treated with fipronil and imidacloprid took minimum time (7.66 and 7.81 h, respectively) to cause 50% mortality, both were statistically at par based on overlapping of 95% CLs, while the fabrics treated with spinosad and indoxacarb took relatively more time to cause 50% mortality (Table 1). Spinosad and indoxacarb are relatively slow acting insecticides [30] that might be the reason for their relatively higher LT_50_ values. The results indicate the potential of four insecticides for use in toxic baits for house flies. The success of toxic baits depends on many factors including visual and/or olfactory lures to attract and ultimately kill flies [31]. The United States Environmental Protection Agency (EPA) has categorized some chemicals used in insecticides as “reduced risk” chemicals based on their effects on environmental health and fate, safety to humans and other animals, and the insecticides tested in the present study are from this category [32]. The combination of toxic baits together with a phagostimulant like sugar, and a visual lure like color, could be effective in eliciting house fly mortality. Previously, sugar has been found to increase the attraction of flies [6,11] and mosquitoes [33,34] towards toxic baits. Based on LT_50_ values, imidacloprid and fipronil could be more effective for such an approach. Imidacloprid is a neonicotinoid with low mammalian and non-target organism toxicity [35]. There is an emerging trend in the use of house fly baits containing imidacloprid in the last few years. In the United States, for example, granular imidacloprid-baits against house flies have been in use in a variety of animal production facilities since 2004 [36]. Moreover, the known tolerance of some insect predators to imidacloprid is advantageous concerning the safety of non-target organisms [37]. Fipronil, a broad spectrum phenylpyrazole, is highly toxic to dipterans including dairy populations of house flies [38], malarial and dengue mosquitoes [32], and fruit flies [39]. Although, fipronil will have a potential to control house flies in the future [38], baits containing fipronil should be evaluated at livestock farms under varying environmental conditions.

**Table 1:** Toxicity of insecticide treated dark blue fabric strips to adult house flies.

Chemical	*n*	LT_50_ * (hour)	Fit of probit line				
		(95% CL)	Slope (SE)	χ^2^	df	P	
Fipronil	120	7.81 (5.97-9.97)^a^	1.52 (0.16)	5.01	3	0.17	
Imidacloprid	120	7.66 (5.73-9.86)^a^	1.39 (0.15)	5.11	3	0.16	
Indoxacarb	120	17.91 (14.78-21.96)^b^	2.06 (0.24)	2.52	3	0.47	
Spinosad	120	13.62 (10.76-17.32)^b^	1.61 (0.18)	4.48	3	0.21	

* LT_50_ = Lethal time to kill 50% population. Confidence limits with similar letters are statistically at par based on non-overlapping of 95% CL.

In conclusion, the combination of phagostimulant and visual lure along with insecticides could be used in designing fly baits. Dark blue was found to be the most attractive to house flies and hence can be used in designing toxic baits. Although the formulated products used are not developed for baits or house flies management, a relative comparison of the products is presented which could be helpful as there is a lack of recommended public health or veterinary pesticides in Pakistan. Since toxic baits reduce the chances of resistance development in flies, these could be potential candidates in developing chemical based management strategies for house flies. However, field evaluation of these baits needs to be done before inclusion in management programs.
